# Extensive erythematous plaques of fungal origin in an overseas student: Cutaneous manifestation of coccidioidomycosis

**DOI:** 10.1016/j.mmcr.2024.100674

**Published:** 2024-10-01

**Authors:** Qi-Hao Yao, Xiu-Jiao Xia, Hui-Lin Zhi, Ze-Hu Liu

**Affiliations:** a Department of Dermatology, Hangzhou Third People's Hospital, West Lake Rd 38, Hangzhou, Zhejiang, 310009, China; b Zhejiang University School of Medicine, Kaixuan Rd 268, Hangzhou, Zhejiang, 310009, China

**Keywords:** Coccidioidomycosis, *Coccidioides posadasii*, Next generation sequencing, Drug sensitivity test, Molecular diagnosis

## Abstract

We present a case of *Coccidioides posadasii* infection which was contracted during study abroad. This coccidioidomycosis showed atypical manifestations and was diagnosed by a combination of tissue biopsy, metagenomic next–generation sequencing, internal transcribed spacer sequencing and culture. Initial treatment with fluconazole was not effective. Antifungal therapy was switched to voriconazole based on drug sensitivity results with good result. This case demonstrates the clinical significance of combining multiple diagnostic methods.

## 1 Introduction

Coccidioidomycosis, a group of endemic mycoses, is caused by the fungi *Coccidioides posadasii* and *Coccidioides immitis* and is predominantly found in arid regions of the Americas [1]. The disease can affect multiple organ systems including the respiratory system, skin and soft tissues, and the central nervous system, with the respiratory system being the most frequently affected [2]. With the increase in international travel and exchanges, cases of coccidioidomycosis have been increasingly reported in non-endemic areas, notably among young adults with intact immune systems. The rarity of coccidioidomycosis in non-endemic regions, coupled with non-specific clinical symptoms, often complicates diagnosis.

We present a case of an imported infection by *C. posadasii*, characterized initially by solely cutaneous manifestations. The patient lacked the typical respiratory symptoms or the presence of eosinophilia, and was successfully managed with voriconazole.

## 2 Case presentation

A 21-year-old man, who had been in good health throughout his three years of study at a college in Arizona, returned to China and presented with a three-month history of pruritic, erythematous plaques on his right upper limb and back. Before the initial visit, the patient exhibited no other symptoms, in particular no respiratory symptoms such as cough or difficulty breathing.

**Fig. 1:**
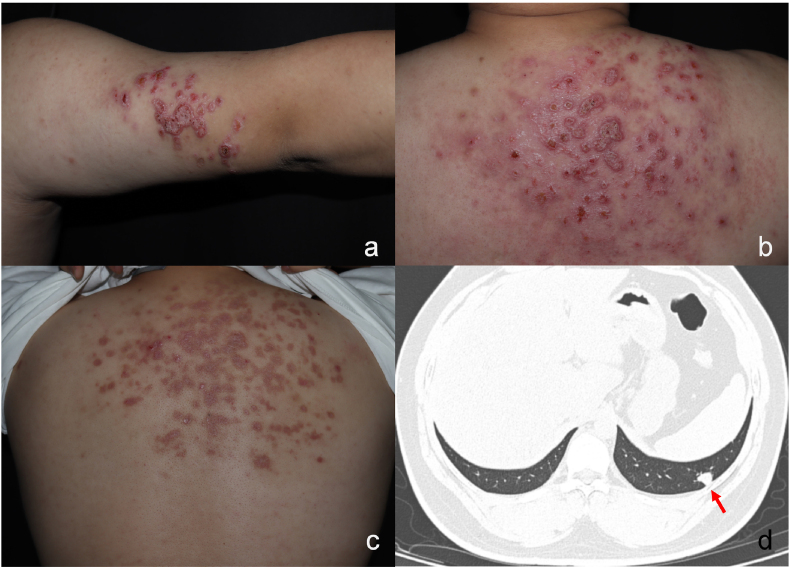
Clinical and imaging presentations of this patient a, b: multiple erythematous plaques with signs of erosion, scabbing, and exudation were observed c: Skin lesions after 6 months of voriconazole treatment, showing improvements; d: Solitary pulmonary nodule found by chest CT with no obvious pulmonary symptoms at presentation.

Physical examination on day 0 showed multiple erythematous plaques with signs of erosion, scabbing, and exudation (Fig. 1a and b). Routine blood tests yielded no abnormal findings. Microscopic examination of skin scrapings and sputum samples on day 0 showed no signs of infection. A chest CT scan detected a solitary nodule in the left lower lobe of the lung (Fig. 1d). Due to the failure of a percutaneous aspiration biopsy of the pulmonary nodule on day 3, the nature of the nodule could not be further defined.

**Fig. 2:**
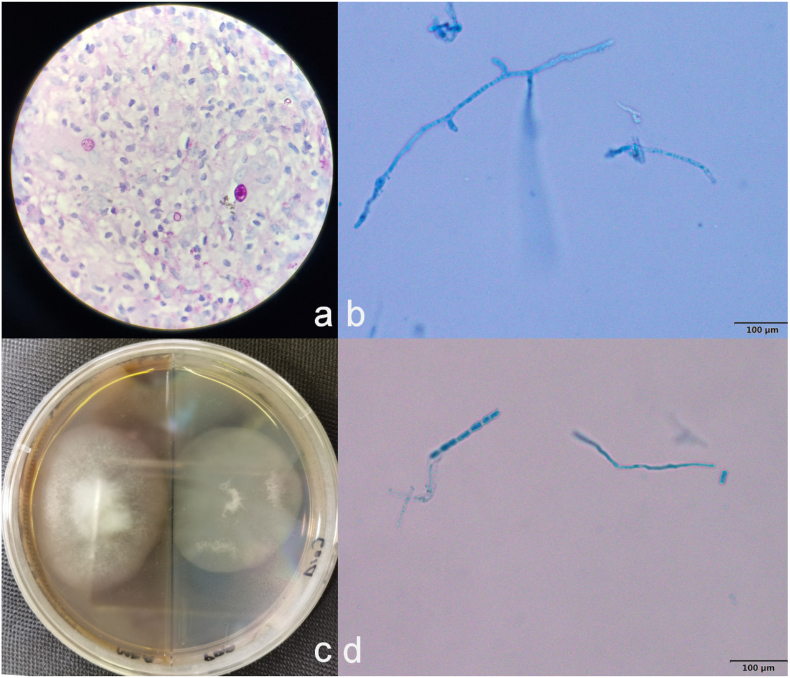
Etiological test results including biopsy and culture a: Round thick-walled spherules of varying size in infectious granuloma ( × 100, in Periodic Acid-Schiff staining); b, d: Slender branched hyphae isolated from the culture (under lactophenol cotton blue staining); c: Grey cotton-like colony produced by 37 °C culture. (For interpretation of the references to colour in this figure legend, the reader is referred to the Web version of this article.)

However, there was a high suspicion for an infectious origin and a biopsy of the cutaneous lesion was sent for histological examination on day 3. Hematoxylin-eosin (HE) and Periodic Acid-Schiff (PAS) staining were performed, and part of the samples were sent for metagenomic next–generation sequencing (mNGS) examination. The results of mNGS suggested the presence of *Coccidioides posadasii* on day 6. The histopathological results were reported on day 10 as follows: HE staining indicated infectious granuloma, with round, thick-walled spherules of varying sizes containing endospores (Fig. 2a) as well as hyphae (Fig. 2b and d) being visible in the PAS staining.

A diagnosis of coccidioidomycosis was made. Given the risk of culturing the relevant strain, culture was conducted at 37 °C in a Biosafety Level 3 (BSL-3) specialized mycology laboratory. The culture showed hoary, cotton-like colonies (Fig. 2c). The identification was based on Internal Transcribed Spacer (ITS) sequencing (GenBank ON 982452.1), showing consistency to *C. posadasii*. The patient was started on fluconazole (400 mg/day) on day 21.

After 3 months of fluconazole treatment, no improvements were observed. An attempt was made to switch to amphotericin B lipid complex IV, but the patient exhibited chills, dyspnea and shock. Therefore, fluconazole treatment was continued for an additional three months, unfortunately without any effect. Drug sensitivity testing was conducted on the strain originally cultured to evaluate the strain's susceptibility to a wide range of antifungal agents showing the minimum inhibitory concentration (MIC) of 8μg/mL for fluconazole and 0.06μg/mL for voriconazole (Table 1). Based on these results, antifungal therapy was changed to voriconazole 300 mg twice daily (increased dose due to patient's overweight). Blood eosinophil counts were repeatedly assessed, and were only once increased (0.7 × 10^9^/L) during the course of the infection. Therapeutic drug monitoring for voriconazole was not performed due to technical limitations. Follow-up was continued once a month to monitor clinical improvement. After 12 months, the skin lesions had subsided (Fig. 1c), and three consecutive mycological tests conducted over a one-year period, including direct microscopic examination of the skin scrapings and culture in, yielded negative results.

**Table 1:** Results of drug sensitivity tests.

	Drug	Concentration μg/mL
Minimum effective concentration (MEC)	anidulafungin	0.03
	micafungin	0.03
	caspofungin	0.03
Minimum inhibitory concentration (MIC)	5-Fluorocytosine	＞64
	posaconazole	0.25
	voriconazole	0.06
	itraconazole	0.5
	amphotericin B	0.5
	fluconazole	8

## 3 Discussion

Coccidioidomycosis, commonly referred to as valley fever or desert fever, is an endemic mycosis affecting regions of the Americas characterized by arid climates, including California and Arizona [1]. The causative agents of coccidioidomycosis are *C. posadasii* and *C. immitis*, which exhibit no significant differences beyond the genetic level, leading to their indistinguishability in clinical practice [3]. Notably, *C. posadasii* is more challenging to isolate from endemic soils compared to *C. immitis* for reasons that remain unclear, thereby complicating associated research efforts [4]. The transformation of *Coccidioides* from mycelium to arthrospores, triggered by climatic shifts, is responsible for the release of spores that, when inhaled or otherwise introduced into a host, can lead to infection [4].

The disease was traditionally believed to be predominantly prevalent in arid regions, such as valleys and deserts in the southwestern United States, with the majority of sporadic cases in China being imported, linked to a history of travel [5]. Nonetheless, recent years have seen an increase in suspected cases of local infection in China with absence of overseas travel history. Most of these cases were associated with immunosuppressive factors: living in an economically underdeveloped area, or having a history of malignancy or immunosuppressive drugs use such as glucocorticoids [6]. Based on geographical, ecological, and meteorological factors in the affected regions, certain scholars have concluded that conditions conducive to the existence of *Coccidioides* spp. are present in some areas of China [6]. Additionally, reports indicate that small rodents are capable of harboring *Coccidioides* spp., with indigenous cases emerging in non-traditional endemic regions within the Americas [7]. These observations suggest that *Coccidioides* possesses environmental adaptability that facilitates migration, warranting further ecological research.

Due to its rarity in non-endemic regions and the non-specific nature of clinical symptoms, the disease is frequently misdiagnosed. The traditional diagnostic methods include mycological culture, histopathological examination and serological examination, but all of them have some disadvantages. Laboratory culture requires specific conditions and prolonged culture period, and need for a biosafety level 3 laboratory [8]. When laboratory exposure to *Coccidioides* occurs, people unfamiliar with the isolate may inhale much higher amounts of arthroconidia than natural exposure, leading to serious infection. This situation can be avoided through a well-developed risk assessment process and preventive measures. In detail, the culture of an unknown mold should be opened in a cabinet with appropriate biological safety, or the isolates should be examined in a BSL-3 laboratory. While delivering samples with *Coccidioides* in sealed containers, the formation of airflow, which results in the formation of aerosols, should be avoided during the subsequent operation. Experimental personnel should strengthen personal protection and waste disposal.

Traditional histopathology relies on the identification of spherules with characteristic endospores, a task that is challenging in cases with atypical presentations or low fungal burden, and demands precise sampling site and depth [9]. Serological testing for coccidioidomycosis in non-endemic regions, like China, is not routinely performed and presents significant challenges for implementation. Emerging molecular techniques, including the sequencing of specific regions within the ITS segment, are increasingly utilized in fungal taxonomy and clinical diagnostics [10]. Next Generation Sequencing (NGS) demonstrates the advantage of detecting low-abundance pathogens without the need for amplification and offers broad coverage in the diagnosis of complex infections [11].

In our case, the patient exhibited predominant skin lesions without significant respiratory symptoms, even with the presence of an isolated pulmonary nodule. Coccidioidomycosis primarily presents as a respiratory disease, characterized by symptoms such as fever, cough, expectoration, hemoptysis, and dyspnea, with primary cutaneous manifestations being less common [2]. Considering the examination results, we hypothesize that the rash may be a secondary manifestation resulting from the dissemination of *C. posadasii*, yet the absence of pronounced pulmonary symptoms remains puzzling.

At the onset, the lack of significant eosinophilia is also uncommon. Eosinophilia is a common feature in numerous fungal infections, including coccidioidomycosis, histoplasmosis, and pulmonary aspergillosis. The precise role of eosinophils in fungal infections is not fully understood, but they may function by phagocytosing pathogens and releasing eosinophil extracellular traps (EET) [12]. Prior research indicates that elevated blood eosinophil levels are a significant diagnostic indicator for coccidioidomycosis [13].

The patient's mild pulmonary symptoms and low blood eosinophil counts certainly complicated the diagnostic process. Consequently, a multifaceted diagnostic approach was employed in this instance. Both conventional (culture, histopathology) and molecular diagnostic tools were performed in parallel and led to the diagnosis of coccidioidomycosis.

The culture with the help of a specialized mycology laboratory clarified the morphology of the colony and provided the material basis for the drug sensitivity tests. The ITS sequencing confirmed the isolate being *C*. *posadasii.*

In terms of treatment, the existing literature advocates for the use of itraconazole and fluconazole in cases of mild to moderate severity, with intravenous amphotericin B in severe or refractory cases [14].

Regrettably, our patient exhibited a poor response to the conventional 6-month fluconazole treatment, with subsequent tests indicating resistance to the drug. In similar cases with poor response to antifungal therapy, a drug sensitivity test should be performed on the original isolate: the results obtained on this isolate might better guide drug change. Additionally, amphotericin B lipid complex was not a viable option due to severe adverse drug reactions. Consequently, we opted for voriconazole as the long-term oral therapy for this patient.

However, therapeutic drug monitoring for voriconazole was not performed due to limitations at the time. Close clinical follow-up was performed with a successful outcome. No adverse events were reported during follow-up. The treatment spanned a total of 18 months, consisting of an initial 6-month course of fluconazole with suboptimal efficacy, followed by 12 months of voriconazole. Treatment was discontinued subsequent to three consecutive negative mycological assessments of direct microscopic examination of the skin scrapings and culture.

In summary, we report a case of coccidioidomycosis in a non-endemic region contracted during study abroad, with significant symptoms only emerging months after the patient's return to China. This case highlights the diversity of manifestations in certain diseases and emphasizes the critical need for a comprehensive diagnostic strategy, especially when a single approach is not sufficient to identify the causative agent or to determine the most effective treatment.

## Conflict of interest

There are none.

## CRediT authorship contribution statement

**Qi-Hao Yao:** Writing – original draft, Visualization, Methodology, Investigation, Formal analysis. **Xiu-Jiao Xia:** Writing – review & editing, Resources, Data curation. **Hui-Lin Zhi:** Writing – review & editing, Resources, Investigation, Conceptualization. **Ze-Hu Liu:** Writing – review & editing, Visualization, Supervision, Methodology, Funding acquisition, Conceptualization.
