# Supplementary material for: Extensive erythematous plaques of fungal origin in an overseas student: Cutaneous manifestation of coccidioidomycosis
Source: Med Mycol Case Rep. 2024 Oct 1;46:100674. doi: 10.1016/j.mmcr.2024.100674 (PMC11489327; doi:10.1016/j.mmcr.2024.100674)
Supplement: Multimedia component 2 [file mmc2.docx]

| Primer name | The number of bases | Primer sequence (5’-3’) |
| --- | --- | --- |
| ITS1F | 19 | TCCGTAGGTGAACCTGCGG |
| ITS4R | 20 | TCCTCCGCTTATTGATATGC |
